# Supplementary material for: β2 Integrins on Dendritic Cells Modulate Cytokine Signaling and Inflammation-Associated Gene Expression, and Are Required for Induction of Autoimmune Encephalomyelitis
Source: Cells. 2022 Jul 13;11(14):2188. doi: 10.3390/cells11142188 (PMC9322999; doi:10.3390/cells11142188)
Supplement: Supplementary file 1 [file cells-11-02188-s001.zip › cells-1761190-supplementary.pdf]

cells

MDPI

Article

β2 integrins on dendritic cells modulate cytokine signaling and inflammation-associated gene expression, and are required for induction of autoimmune encephalomyelitis

Monika Bednarczyk[1], Vanessa Bolduan[1], Maximilian Haist[1], Henner Stege[1], Christoph Hieber[1], Lisa Johann[2], Carsten Schelmbauer[2], Michaela Blanfeld[2], Simone Wörtge[2], Khalad Karram[2,3], Jenny Schunke[1], Tanja Klaus[1], Ingrid Tubbe[1], Evelyn Montermann[1], Nadine Röhrig[1], Maike Hartmann[1], Jana Schlosser[1], Tobias Bopp[3,4], Björn E. Clausen[2,3], Ari Waisman[2,3], Matthias Bros[1,3] and Stephan Grabbe[1,3,*]

1 Department of Dermatology, University Medical Center of the Johannes Gutenberg-University Mainz, 55131 Mainz, Germany, m.bednarczyk.09@aberdeen.ac.uk (M.Be.), vbolduan@students.uni-mainz.de (V.B.), mhaist@uni-mainz.de (M.Ha.), Henner.Stege@unimedizin-mainz.de (H.S.), C.Hieber@imb-mainz.de (C.H.), jschunke@students.uni-mainz.de (J.S.), tklaus@students.uni-mainz.de (T.K.), tubbe@uni-maniz.de (I.T.), monterma@uni-mainz.de (E.M.), n.roehrig@uni-mainz.de (N.R.), Maike.Hartmann@unimedizin-mainz.de (M.Har.), schlosser.jana@stud.hs-fresenius.de (J.S.), mbros@uni-mainz.de (M.Br.), stephan.grabbe@unimedizin-mainz.de (S.G.)

2 Institute for Molecular Medicine, University Medical Center of the Johannes Gutenberg University of Mainz, Mainz, , Langenbeckstraße 1, 55131 Mainz, Germany; lijohann@students.uni-mainz.de (L.J.), cschelmb@uni-mainz.de (C.S.), blanfeld@uni-mainz.de (M.Bl.), karram@uni-mainz.de (K.K.), bclausen@uni-mainz.de (B.E.C.), waisman@uni-mainz.de (A.W.)

3 Research Center for Immunotherapy (FZI) University Medical Center of the Johannes Gutenberg University of Mainz, Mainz, , Langenbeckstraße 1, 55131 Mainz, Germany; karram@uni-mainz.de (K.K.), boppt@uni-mainz.de (T.B.), bclausen@uni-mainz.de (B.E.C.), waisman@uni-mainz.de (A.W.), mbros@uni-mainz.de (M.Br.), stephan.grabbe@unimedizin-mainz.de (S.G.)

4 Institute of Immunology, University Medical Center of the Johannes Gutenberg University Mainz, Langenbeckstraße 1, boppt@uni-mainz.de (T.B.)

*Correspondence: stephan.grabbe@unimedizin-mainz.de; Tel.: +49 6131 17 4412

LOCUS B044.2 13010 bp DNA circular SYN 07-DEC-2012

DEFINITION B044.2 rotated to position 10485

ACCESSION B044.2

REFERENCE 1 (bases 1 to 13010)

AUTHORS Self

JOURNAL Unpublished.

COMMENT SECID/File created by Clone Manager, Scientific & Educational Software

FEATURES Location/Qualifiers

CDS 1..2451

/gene="'100% B6'"

	/codon_start=3
	/SECDrawAs="Gene"
	/SECStyleId=1
Misc._feature	1..2451
	/gene="SA"
	/SECDrawAs="Region"
	/SECStyleId=1
	/label="Misc. feature"
CDS	complement(1693..1727)
	/gene="loxP"
	/SECDrawAs="Gene"
	/SECStyleId=1
CDS	2106..2286
	/gene="Ex 3"
	/SECDrawAs="Gene"
	/SECStyleId=1
CDS	2555..2602
	/gene="FRT"
	/SECDrawAs="Gene"
	/SECStyleId=1
CDS	complement(3097..3900)
	/gene="neo"
	/SECDrawAs="Gene"
	/SECStyleId=1
CDS	4473..4520
	/gene="FRT"
	/SECDrawAs="Gene"
	/SECStyleId=1

Misc._feature	4501..8671
	/gene="LA"
	/SECDrawAs="Region"
	/SECStyleId=1
	/label="Misc. feature"
CDS	complement(4601..4635)
	/gene="loxP"
	/SECDrawAs="Gene"
	/SECStyleId=1
CDS	5424..5594
	/gene="Ex 4"
	/SECDrawAs="Gene"
	/SECStyleId=1
CDS	6844..7085
	/gene="Ex 5"
	/SECDrawAs="Gene"
	/SECStyleId=1
CDS	8244..8399
	/gene="Ex 6"
	/SECDrawAs="Gene"
	/SECStyleId=1
Misc._feature	8662..10456
	/gene="TK"
	/SECDrawAs="Region"
	/SECStyleId=1
	/label="Misc. feature"
CDS	8963..9892
	/gene="TK"

/SECDrawAs="Gene"

/SECStyleId=1

ORIGIN

```
   1 CCGGTGTCCC TCTGCTAGTG GTGTTTCCGG AGGTGCCACC TTCCAGATTT CTCCTCACTT
  61 CCTATGTCAC CTTATAGTGA GTGACAGGCC ATCAGTGAGG CTCTATCAAG CTGGCTGTCT
 121 CCACTAAACT ATATCTGTGC CCTTGCAGTC TGTCTACTGA AGACTCAGGC AGGATCCATT
 181 ACCAATGTAG TGGTTATTAA ATGGCTCTCT GCCTCTCTCT CTCTCTGTCT CTCTCTGTCT
 241 TTCTGTCTCT CTGTCTCTCG TGTGTGTGTG TGTGTGTGTG TGTGTGTGTG TGTGTGTCTC
 301 CAGACTTCTA CCTCCATTGG TAAATCTGGA GATCTTAATT CTGGTCAGTA GTTTATAACC
 361 ATTATTTTTC CAATCTGATG TTCAAGCTGT CCACATCTGG CCGCTGGACC CCTCTAAGCT
 421 GTTCTGGTGT TCTGGGTGCT GTTCATATGA GCTTTCTTGT CATTTTATTT TGTCCTCAAA
 481 TTTTTCTTAC TCTAGACTGA CTAGCTGTGC TGACTGTACT CTGGCCTCCC CTCCCCTCGC
 541 CCAAGAGTTA GAGTTAAAGA CCACTCCTAG TATTGTGTTT TATAAAACGA TAGGGAGAAA
 601 AGAAGTGTGT TTGCTGGATG TACAGTCAGG TGTGGGAAGG GGGTGCCTCT GCAGGCCCAG
 661 GCTGAGGCAT CCCTTCCCCC TGAGGGACCA GCCACACGAT GGTATAGTAT AGAATAAAGT
 721 TTGTTCAGGG CATGGGGAGG GGGTTTAAGG GAGCAGAGGT GGATAAGGGC AGAGAGAGAG
 781 AGGGAGAGAG AGAGAGAGAG GGAGAGAGAG AGAGAGAGAG AGAGAGAGAG AGAGAGAGAG
 841 AGAGAGAATA TGAATAGTAG TAGAGGTCAG CTATGAGCAC ATGGAGCATG GGTTAAGGAG
 901 CAGGAGCAAG GGCAAGAGAG AGGAAGGAGT AAGCAGCCTC CTTCGTAGTG AGTCAGGCAC
 961 ACCTGGCTAT TGCCAGGTAA CTGTGGGGTG GAGCCTAGAC TAAATGCCAA CTTCTAGGCC
1021 TTCGCATTCT GTGCTACTTT TCTCATCTTC TCCATCCCAC AAGGCTTCTG GCTTCCATCT
1081 CCAGCAAACA CCCCCACCGT AGGCAGAGCT CCAGAGCAGA AAGGCCTGAT GTTGTAACCT
1141 CTATCCTGTG ACTGAGGGCT GTGGTCCAAG CCTTGGACGA GCCATTCTCA GATGAGCTGG
1201 CGTTTCTCTC TTCCTCTTGA GTCATTTGGC TCATCTGAAA TGAACAGCCA GATGGCTAGT
1261 TCTGCATGGA TTACCAGTTA GCTTCTCTTC TCTCTGTTCT CTCTGGTTGC TTGTGTTTCT
1321 TGAGGACTTA ACACGGGGCT CTGAAGGCCA ATGCAGAACA GAGGGACTCT GGAGGTGATG
1381 TCTACTGCAT TTCTGGGTTG GTGTTCTTCT GGGTTTACTT TCTGTGAGCA AGCAGGCAAT
1441 CCCTGGTGCT TTCCAGTACC TTGCAGTTGA GCCAGACCTG CTTTGGATGC AATGGGTTCC
```

1501 AGTTCAGGAC CAAGAACAAC CAAATACTTC CATGTCAGAT CCATGGGACC TTTGAAGGGA

1561 GATGGTGGTC AAGATGGAGG TGCCCTGAGC TCTCCAGTCT CCTCACCAAC TCCATTCCCT

1621 ATGAACCGAG AGAGACTTGA CCCCTAGATC TTCCCTGCAT CTCAGCAAGA GCTGTCACAT

1681 AAGCCTAAAG AGATAACTTC GTATAATGTA TGCTATACGA AGTTATACGC GTCACCTTTG

1741 GAGATGGCAT CTCCCCGGCA TCTCAACAGA CAAAGGCTCT GTTGAGAAAT GGTTAAAGAG

1801 TTCTTCTGGT CAACACTGAA ACTCAAGGAT GCTGATTAGC CTTTTGATTG GAGGTAGAGG

1861 ACCCCATGTC TTTGCTGATG AAGTCAGTGC CCAAATGATA GAGAAAGGTC TATATAGTCT

1921 ATGCTTTTGG TAACACAGAG CTCTCGGGGA GACCTAGACT CTCTGGGAGT CCGGTCTTCC

1981 TTAGATGACT ACCGCATCCC ACAGAGATGT CTAACTGAAC TTTCAAGGGC AGGTAAGAAG

2041 GAAACAGGGC TGGATCCCAC TATTCTAGCC TATGCCCTCT GCCTGACCTC AGATCCCTCC

2101 TCTAGAACTT CACTGGACCA GGAGAACCTG ACTCCTTGCG CTGTGACACA CGAGCACAGC

2161 TGCTGCTGAA GGGTTGTCCA GCCGATGATA TCATGGACCC CAGGAGCATC GCTAATCCTG

2221 AGTTCGACCA ACGGGGGCAA CGGAAACAGC TATCTCCACA AAAAGTGACA CTTTACTTGC

2281 GACCAGGTAG GTATGGAACC TGGTTGGAAT GTGTGTGTGT GTGTGGCGGG GGGGGGGGGG

2341 GGTGGAACAT CCCAGAGGGT AGGGAGAAGA ACCATGGCCA TTGACATGAA CCCGCATGGC

2401 AATTACCAGC CACAAGTTGC TATGATTTCA TAAGGTTAGA AATATTGGGT TGTAATACGA

2461 CTCACTAGTG GGCAGATCTT CGAATGCATC GCGCGCACCG TACGTCTCGA GGAATTCCTG

2521 CAGGATTTAA GGCCTTGACT AGAGGGTACC TCGAGAAGTT CCTATTCCGA AGTTCCTATT

2581 CTCTAGAAAG TATAGGAACT TCGGATAATT CTAGAGTCAG CTTCTGATGG AATTAGAACT

2641 TGGCAAAACA ATACTGAGAA TGAAGTGTAT GTGGAACAGA GGCTGCTGAT CTCGTTCTTC

2701 AGGCTATGAA ACTGACACAT TTGGAAACCA CAGTACTTAG AACCACAAAG TGGGAATCAA

2761 GAGAAAAACA ATGATCCCAC GAGAGATCTA TAGATCTATA GATCATGAGT GGGAGGAATG

2821 AGCTGGCCCT TAATTTGGTT TTGCTTGTTT AAATTATGAT ATCCAACTAT GAAACATTAT

2881 CATAAAGCAA TAGTAAAGAG CCTTCAGTAA AGAGCAGGCA TTTATCTAAT CCCACCCCAC

2941 CCCCACCCCC GTAGCTCCAA TCCTTCCATT CAAAATGTAG GTACTCTGTT CTCACCCTTC

3001 TTAACAAAGT ATGACAGGAA AAACTTCCAT TTTAGTGGAC ATCTTTATTG TTTAATAGAT

3061 CATCAATTTC TGCAGACTTA CAGCGGATCG ATCCCCTCAG AAGAACTCGT CAAGAAGGCG

3121 ATAGAAGGCG ATGCGCTGCG AATCGGGAGC GGCGATACCG TAAAGCACGA GGAAGCGGTC

3181 AGCCCATTCG CCGCCAAGCT CTTCAGCAAT ATCACGGGTA GCCAACGCTA TGTCCTGATA

3241 GCGGTCCGCC ACACCCAGCC GGCCACAGTC GATGAATCCA GAAAAGCGGC CATTTTCCAC

3301 CATGATATTC GGCAAGCAGG CATCGCCATG GGTCACGACG AGATCATCGC CGTCGGGCAT

3361 GCGCGCCTTG AGCCTGGCGA ACAGTTCGGC TGGCGCGAGC CCCTGATGCT CTTCGTCCAG

3421 ATCATCCTGA TCGACAAGAC CGGCTTCCAT CCGAGTACGT GCTCGCTCGA TGCGATGTTT

3481 CGCTTGGTGG TCGAATGGGC AGGTAGCCGG ATCAAGCGTA TGCAGCCGCC GCATTGCATC

3541 AGCCATGATG GATACTTTCT CGGCAGGAGC AAGGTGAGAT GACAGGAGAT CCTGCCCCGG

3601 CACTTCGCCC AATAGCAGCC AGTCCCTTCC CGCTTCAGTG ACAACGTCGA GCACAGCTGC

3661 GCAAGGAACG CCCGTCGTGG CCAGCCACGA TAGCCGCGCT GCCTCGTCCT GCAGTTCATT

3721 CAGGGCACCG GACAGGTCGG TCTTGACAAA AAGAACCGGG CGCCCCTGCG CTGACAGCCG

3781 GAACACGGCG GCATCAGAGC AGCCGATTGT CTGTTGTGCC CAGTCATAGC CGAATAGCCT

3841 CTCCACCCAA GCGGCCGGAG AACCTGCGTG CAATCCATCT TGTTCAATGG CCGATCCCAT

3901 ATTGGCTGCA GGTCGAAAGG CCCGGAGATG AGGAAGAGGA GAACAGCGCG GCAGACGTGC

3961 GCTTTTGAAG CGTGCAGAAT GCCGGGCCTC CGGAGGACCT TCGGGCGCCC GCCCCGCCCC

4021 TGAGCCCGCC CCTGAGCCCG CCCCCGGACC CACCCCTTCC CAGCCTCTGA GCCCAGAAAG

4081 CGAAGGAGCA AAGCTGCTAT TGGCCGCTGC CCCAAAGGCC TACCCGCTTC CATTGCTCAG

4141 CGGTGCTGTC CATCTGCACG AGACTAGTGA GACGTGCTAC TTCCATTTGT CACGTCCTGC

4201 ACGACGCGAG CTGCGGGGCG GGGGGGAACT TCCTGACTAG GGGAGGAGTA GAAGGTGGCG

4261 CGAAGGGGCC ACCAAAGAAC GGAGCCGGTT GGCGCCTACC GGTGGATGTG GAATGTGTGC

4321 GAGGCCAGAG GCCACTTGTG TAGCGCCAAG TGCCCAGCGG GGCTGCTAAA GCGCATGCTC

4381 CAGACTGCCT TGGGAAAAGC GCCTCCCCTA CCCGGTAGAA TTGACCTGCA GGGGCCCTCG

4441 ATATCAAGCT CCTTGACTAG AGGGTACCTC GAGAAGTTCC TATTCCGAAG TTCCTATTCT

4501 CTAGAAAGTA TAGGAACTTC AGATCTGGCC TCGTGGGGCA GGTAGTGGGG AGAGCCTGAT

4561 AGTTTTCCTT ACTGTGTGCA ATGTTTTCCC ACAGGACAAC ATAACTTCGT ATAATGTATG

4621 CTATACGAAG TTATAAGCTT TATCATTATC AACTGGCTCT GAAATTCTTT ATTGGCATCT

4681 TGTAAATTGT TATTTTATTG ATACATAAAT CTAATTGGTT AACTTTAAGA TTAAGAGTCT

4741 AATTTTGCTT GGCAGTGGTG GCACATGCCT TTAATCCCAG CACTTAGGAG GCAGAGGCAG

4801 ATGGATTTCT GAGTTCAAGG CCAGCCTGGT CTACAGAGTG AGTTCCAGGA CAGCCTGGGC

4861 TATACAGAGA AACCCTATTT CAAAAAGCCA AAAAAAAAAA AAAGTCTGAT TTTACCAGAG

4921 TATTGGGTGT TGTGAGATGC AACTGCGAGG TTGGTGGTTC TATTTGGCCA CTGGAGTGTG

4981 GGATCTCTGG TTACAGGGGA TTATAGCAGA AAGGGAACTA GTGGTTCCCT GGACAAGCAA

5041 GCCAGATGCT GCAGGTGGGG GGGGGGCAGG TAGCTGCAGA GAGTTGACAG GTTCATTTTT

5101 TAATATTTCC ACAATAGATG GCACCCAATG TGGGGCACCT GACTATTTCT AGCACCATAA

5161 TCTGGGTGTC TAGGCTTCCA ACTCATGAAT CTGCACCTTA CAGAAACCCT CCACCATCCA

5221 GGCATTCTTG TACCTGGGGT GAGCCTACTT ATCTGAGTTC AAACCTACCC TTAAGAAGCT

5281 TTACGTAAGT ACTTGTCCTG TGTTCTGGTC ACAACCCCTT CCAGGTCATA GGAAATGGGG

5341 GAGACAATAG GAATCTGGGG ACTGGGTGAG AGCCAAGACT CCAGCCAGGC TCAGAGTGTC

5401 CCTTGCTTTT CTCTGACACG CAGGACAGGC TGCCGCATTC AATGTGACTT TCCGGCGGGC

5461 CAAGGGATAC CCCATTGATC TGTACTACCT CATGGATCTC TCCTACTCCA TGCTTGATGA

5521 CCTCAACAAC GTCAAGAAGC TGGGCGGGGA CTTGCTGCAG GCCCTCAACG AGATCACCGA

5581 GTCTGGCCGC ATCGGTGAGA CCTGCAGTGC ACACACTCCT TTCAACCTTG GGAACTCCCT

5641 GCCGCCCCAC ACCTCCATGT ACGGTGGATC CGCAGAGGCA GAAGCTGGGA GCTGCATAGG

5701 CAATGTTCGT GGGGCCAGAG TTCATCCTGA ATCCTTACCA GGGGCTGTCC TTGGTCACAG

5761 GCTGCTAAGA CCTCTCTCTC TCTCTCTCTA GTTTATATTT TAATGTTTAA AAGGCAAAAG

5821 TCCTACAATG CTAATTACTA AAAAGTATAT CTACCTTAAC ACAGCAACCA TGCATTAACA

5881 GAATAGTGGA AGTAAACTAC AGGGCAGGGG TCACAGGGCT AGGGCCACTG GGCAAGGGTC

5941 ACAGGGCAGG AGTCATAGGG TGAGAATTAC AACACTGACC AATACCACCA AGCATGTGGG

6001 ACAAGGGAAC AGGGTACACC TACTCACTCC AGAACATCCC TCTGCCTCAT CGTCTATGTT

6061 TAAGGATGCC CACATGAACA CGAACACTGG ATGCAAGCTC ATATATTCTT CTTTTTTTTT

6121 TTTAAGACTT TTTAATTTCA TTTATATGAG TACACTGTAG CTGTCTTCAG ACACACCCGA

6181 AGAGGGCATC AAATCCCATG ACAGATGGTT GTGAGCCACC ATGTGGTTGC TGGGAATTGA

6241 ACTCAGAACC TCTGAAAGAG CAGTCAGTGC TCTTACCCGC TGAGCCATCT CTCCAGCCCC

6301 CATATATTCT TGTGTGTTCA TACATTCACA TGTGTACATT CGTGTTCACA CATGTCCAAG

6361 ATCCCATACA CAAGCACATT TGCACATGTG CAAGCAAGCA CATGTACAAA TGCCTAGTCA

6421 TACCTGTGTG CCTGTATGCC TGCATACACA AGACCACATG TATATATACA GGTACTCGCA

6481 TGCCCACCCA CACTCATGGT CACACCTGTC CATGACCCCA TACATATACA TGCTCTGTCA

6541 CATGCACACA CATGTTTGTA CATGGCCTCA CATATACTCA GCCGCACATG GCTACACACA

6601 TGCTTGCACC ACACGCTCAG CTATCATGTT CACACAACCA TCATCTTGCT TTCTCCCTTG

6661 TTTGAGAAAC TGAGGCTTGG TCCTTAGTCC ACTGGAAGGT TCACTGTCAT GCCTGGAGCC

6721 CTGCCTGCTG CCTTGTCCCT GTGCCCTCCA GCCTCTGTTG TATATCCCTT GGACTCCAGG

6781 TCTTCCCAGT CCTGGTGTCT GCTTCTGTCT GCTGTCCCCC TGCCTCATAC CATGTACCCT

6841 TAGGCTTTGG GTCGTTTGTG GACAAGACGG TGCTGCCTTT TGTTAACACC CATCCTGAGA

6901 AGCTGAGGAA CCCATGTCCC AACAAGGAGA AGGCCTGCCA GCCCCCATTT GCCTTTCGGC

6961 ACGTGCTCAA GTTAACCGAC AACTCCAACC AGTTTCAGAC AGAGGTCGGC AAGCAACTGA

7021 TTTCCGGAAA CCTGGACGCC CCTGAGGGTG GGCTGGATGC CATAATGCAA GTTGCTGCAT

7081 GTCCGGTGAG GCCATTGTCC CTGTTCAGAT TTCCCAACTT CCTCAAACTC AGGGCCTTCA

7141 CCAGTGCCGT GGTGGTGGGT CTGTTCAGAC ACTCACCCCA TCTGGTCCCA TGCTGTCACC

7201 CCGGGTCTCA GAAGGGCAGC CTCCGTGGGC CACATCACTC CCAGGCATAG AGAAGGAAGA

7261 GCCCTGAAAA CACCCACCCA TGCCCGTGCT AACTGACAGC CTTCATAGCT CTCCTGTGAG

7321 CCAGGCTACC CAGCAGCAGG TCTTCTGGTT CCTAAAGCCT TTTCCCTCTC AGATCAAGAG

7381 TGTGAGAACT GTTGTGGGAC AAAGGAACAG GGGCCCATGT CACCTCAATC ACATCCTGCC

7441 TGTCGGCTCC AGATTGTGTT TATCAAGTGG TCACTGCAGC AAGCCAATCC CACCCCAGCC

7501 TGGCTCTCAG CTCTCTCACT TTGCTGGGTT CACGCAGTGG GCACACACAC TCTACATGTG

7561 TGTTGGACTA GGGTAGGATA CATGGGTGCA GCAAGTGTAT GCTACTTCAA TGGTTCCAGG

7621 CCCTCGGCTC TCCTGTGGAT GTCCCTTGGT CCCTTCTTTT GTGGCTGGGA GGTGTTTGCA

7681 TTTTAACATA CAAGGCCATA CTCTTTTCCT TTCATGACTG TGTTGCTGGA ATCTAGTTTC

7741 AGGTCTGAAG AGGGTTGCAA AGACATTTCT CTTTAGATTC AGATGTGTTT CTGTTCTTAC

7801 CTCATACTGC TGCAGGGCAT GCTGGGAGTT GACGGCTTTG GCACTTCCTT GGCACTGGAT

7861 TGGGGCTCTA TCTTCCTCTG TCTGTCTGTC TGTCTGTCTG TCTGTCTGTC TGTCTGTCTG

7921 TCGTATCTGG GGGGTGTCTG TTTGTTTCCA TGTTTCTGTC TCTCTTTATA TATATATATC

7981 TGTCTCTCTC TTTATCTCTA TCTGTTTTTA TGTCTTGTTG TCTCTGTCTC TATGTCTCTG

8041 TGTCTCTATC ACTCTGTGTG TGTGTGTCTC TTTGTATGTA TGTCTCTGTC TGTTTGTCTG

8101 TCTCTTTCTC TGTCTTTCTA TCTCTCTGTG TCTCTCTTTC TGTCTCTGTC TCTCTCACTC

8161 TCCCTCACAC ACACACAACT CTTCTCCCCT GAGGGCCCCA GGGGGAAACC AAGGCAGGTA

8221 ACCCCTGGAT GCCTTCTTCC CAGGAGGAAA TTGGCTGGCG CAATGTCACG AGGCTGCTGG

8281 TGTTTGCCAC AGACGATGGC TTCCACTTTG CTGGTGATGG CAAACTGGGT GCCATCCTGA

8341 CCCCCAATGA TGGCCGCTGC CACCTGGAGG ATAACATGTA CAAGAGGAGC AATGAGTTCG

8401 TGAGTGCTCT GCCTGCCTTG GCACGCCCCG GTACCAAGGC TGGTTACATA AACATAAAGG

8461 TTACAGGAGT GACAACGGGA CAGGCTGAGA GCAGAGTCAC ATTGCCCAAT CACACAAACA

8521 GAACCCAGAC TCCTCCTAAC ACGAATAACA AATCACAGAT GCAGTCCCAG AGACCCTGGT

8581 GATAACATGA GACTCCCAGC ACCCACATCA GCCTGCAGGG CAGTCCTCAC CCTGATCCTC

8641 ATGGCAGAGG GCCTCACACA GGCGCGCCTC GAGCAGTGTG GTTTTGCAAG AGGAAGCAAA

8701 AAGCCTCTCC ACCCAGGCCT GGAATGTTTC CACCCAATGT CGAGCAAACC CCGCCCAGCG

8761 TCTTGTCATT GGCGAATTCG AACACGCAGA TGCAGTCGGG GCGGCGCGGT CCGAGGTCCA

8821 CTTCGCATAT TAAGGTGACG CGTGTGGCCT CGAACACCGA GCGACCCTGC AGCGACCCGC

8881 TTAACAGCGT CAACAGCGTG CCGCAGATCT TGGTGGCGTG AAACTCCCGC ACCTCTTCGG

8941 CCAGCGCCTT GTAGAAGCGC GTATGGCTTC GTACCCCGGC CATCAACACG CGTCTGCGTT

9001 CGACCAGGCT GCGCGTTCTC GCGGCCATAG CAACCGACGT ACGGCGTTGC GCCCTCGCCG

9061 GCAGCAAGAA GCCACGGAAG TCCGCCCGGA GCAGAAAATG CCCACGCTAC TGCGGGTTTA

9121 TATAGACGGT CCCCACGGGA TGGGGAAAAC CACCACCACG CAACTGCTGG TGGCCCTGGG

9181 TTCGCGCGAC GATATCGTCT ACGTACCCGA GCCGATGACT TACTGGCGGG TGCTGGGGGC

9241 TTCCGAGACA ATCGCGAACA TCTACACCAC ACAACACCGC CTCGACCAGG GTGAGATATC

9301 GGCCGGGGAC GCGGCGGTGG TAATGACAAG CGCCCAGATA ACAATGGGCA TGCCTTATGC

9361 CGTGACCGAC GCCGTTCTGG CTCCTCATAT CGGGGGGGAG GCTGGGAGCT CACATGCCCC

9421 GCCCCCGGCC CTCGCCCTCA TCTTCGACCG CCATCCCATC GCCGCCCTCC TGTGCTACCC

9481 GGCCGCGCGG TACCTTATGG GCAGCATGAC CCCCCAGGCC GTGCTGGCGT TCGTGGCCCT

9541 CATCCCGCCG ACCTTGCCCG GCACCAACAT CGTGCTTGGG GCCCTTCCGG AGGACAGACA

9601 CATCGACCGC CTGGCCAAAC GCCAGCGCCC CGGCGAGCGG CTGGACCTGG CTATGCTGGC

9661 TGCGATTCGC CGCGTTTACG GGCTACTTGC CAATACGGTG CGGTATCTGC AGTGCGGCGG

9721 GTCGTGGCGG GAGGACTGGG GACAGCTTTC GGGGACGGCC GTGCCGCCCC AGGGTGCCGA

9781 GCCCCAGAGC AACGCGGGCC CACGACCCCA TATCGGGGAC ACGTTATTTA CCCTGTTTCG

9841 GGCCCCCGAG TTGCTGGCCC CCAACGGCGA CCTGTATAAC GTGTTTGCCT GAGCCTTGGA

9901 CGTCTTGGCC AAACGCCTCC GTTCCATGCA CGTCTTTATC CTGGATTACG ACCAATCGCC

9961 CGCCGGCTGC CGGGACGCCC TGCTGCAACT TACCTCCGGG ATGGTCCAGA CCCACGTCAC

10021 CACCCCCGGC TCCATACCGA CGATATGCGA CCTGGCGCGC ACGTTTGCCC GGGAGATGGG

10081 GGAGGCTAAC TGAAACACGG AAGGAGACAA TACCGGAAGG AACCCGCGCT ATGACGGCAA

10141 TAAAAAGACA GAATAAAACG CACGGGTGTT GGGTCGTTTG TTCATAAACG CGGGGTTCGG

10201 TCCCAGGGCT GGCACTCTGT CGATACCCCA CCGAGACCCC ATTGGGGCCA ATACGCCCGC

10261 GTTTCTTCCT TTTCCCCACC CCACCCCCCA AGTTCGGGTG AAGGCCCAGG GCTCGCAGCC

10321 AACGTCGGGG CGGCAGGCCC TGCCATAGCC ACTGGCCCCG TGGGTTAGGG ACGGGGTCCC

10381 CCATGGGGAA TGGTTTATGG TTCGTGGGGG TTATTATTTT GGGCGTTGCG TGGGGTCAGG

10441 TCCACGACCC AAGCTGGCGC GCCTACGTAA TCTTGAATCG AATTCCCGCA ACTACGTCAG

10501 GTGGCACTTT TCGGGGAAAT GTGCGCGGAA CCCCTATTTG TTTATTTTTC TAAATACATT

10561 CAAATATGTA TCCGCTCATG AGACAATAAC CCTGATAAAT GCTTCAATAA TATTGAAAAA

10621 GGAAGAGTAT GAGTATTCAA CATTTCCGTG TCGCCCTTAT TCCCTTTTTT GCGGCATTTT

10681 GCCTTCCTGT TTTTGCTCAC CCAGAAACGC TGGTGAAAGT AAAAGATGCT GAAGATCAGT

10741 TGGGTGCACG AGTGGGTTAC ATCGAACTGG ATCTCAACAG CGGTAAGATC CTTGAGAGTT

10801 TTCGCCCCGA AGAACGTTCT CCAATGATGA GCACTTTTAA AGTTCTGCTA TGTGGCGCGG

10861 TATTATCCCG TGTTGACGCC GGGCAAGAGC AACTCGGTCG CCGCATACAC TATTCTCAGA

10921 ATGACTTGGT TGAGTACTCA CCAGTCACAG AAAAGCATCT TACGGATGGC ATGACAGTAA

10981 GAGAATTATG CAGTGCTGCC ATAACCATGA GTGATAACAC TGCGGCCAAC TTACTTCTGA

11041 CAACGATCGG AGGACCGAAG GAGCTAACCG CTTTTTTGCA CAACATGGGG GATCATGTAA

11101 CTCGCCTTGA TCGTTGGGAA CCGGAGCTGA ATGAAGCCAT ACCAAACGAC GAGCGTGACA

11161 CCACGATGCC TGTAGCAATG GCAACAACGT TGCGCAAACT ATTAACTGGC GAACTACTTA

11221 CTCTAGCTTC CCGGCAACAA TTAATAGACT GGATGGAGGC GGATAAAGTT GCAGGACCAC

11281 TTCTGCGCTC GGCCCTTCCG GCTGGCTGGT TTATTGCTGA TAAATCTGGA GCCGGTGAGC

11341 GTGGGTCTCG CGGTATCATT GCAGCACTGG GGCCAGATGG TAAGCCCTCC CGTATCGTAG

11401 TTATCTACAC GACGGGGAGT CAGGCAACTA TGGATGAACG AAATAGACAG ATCGCTGAGA

11461 TAGGTGCCTC ACTGATTAAG CATTGGTAAC TGTCAGACCA AGTTTACTCA TATATACTTT

11521 AGATTGATTT ACCCCGGTTG ATAATCAGAA AAGCCCCAAA AACAGGAAGA TTGTATAAGC

11581 AAATATTTAA ATTGTAAACG TTAATATTTT GTTAAAATTC GCGTTAAATT TTTGTTAAAT

11641 CAGCTCATTT TTTAACCAAT AGGCCGAAAT CGGCAAAATC CCTTATAAAT CAAAAGAATA

11701 GCCCGAGATA GGGTTGAGTG TTGTTCCAGT TTGGAACAAG AGTCCACTAT TAAAGAACGT

11761 GGACTCCAAC GTCAAAGGGC GAAAAACCGT CTATCAGGGC GATGGCCCAC TACGTGAACC

11821 ATCACCCAAA TCAAGTTTTT TGGGGTCGAG GTGCCGTAAA GCACTAAATC GGAACCCTAA

11881 AGGGAGCCCC CGATTTAGAG CTTGACGGGG AAAGCGAACG TGGCGAGAAA GGAAGGGAAG

11941 AAAGCGAAAG GAGCGGGCGC TAGGGCGCTG GCAAGTGTAG CGGTCACGCT GCGCGTAACC

12001 ACCACACCCG CCGCGCTTAA TGCGCCGCTA CAGGGCGCGT AAAAGGATCT AGGTGAAGAT

12061 CCTTTTTGAT AATCTCATGA CCAAAATCCC TTAACGTGAG TTTTCGTTCC ACTGAGCGTC

12121 AGACCCCGTA GAAAAGATCA AAGGATCTTC TTGAGATCCT TTTTTTCTGC GCGTAATCTG

12181 CTGCTTGCAA ACAAAAAAAC CACCGCTACC AGCGGTGGTT TGTTTGCCGG ATCAAGAGCT

12241 ACCAACTCTT TTTCCGAAGG TAACTGGCTT CAGCAGAGCG CAGATACCAA ATACTGTTCT

12301 TCTAGTGTAG CCGTAGTTAG GCCACCACTT CAAGAACTCT GTAGCACCGC CTACATACCT

12361 CGCTCTGCTA ATCCTGTTAC CAGTGGCTGC TGCCAGTGGC GATAAGTCGT GTCTTACCGG

12421 GTTGGACTCA AGACGATAGT TACCGGATAA GGCGCAGCGG TCGGGCTGAA CGGGGGGTTC

12481 GTGCACACAG CCCAGCTTGG AGCGAACGAC CTACACCGAA CTGAGATACC TACAGCGTGA

12541 GCTATGAGAA AGCGCCACGC TTCCCGAAGG GAGAAAGGCG GACAGGTATC CGGTAAGCGG

12601 CAGGGTCGGA ACAGGAGAGC GCACGAGGGA GCTTCCAGGG GGAAACGCCT GGTATCTTTA

12661 TAGTCCTGTC GGGTTTCGCC ACCTCTGACT TGAGCGTCGA TTTTTGTGAT GCTCGTCAGG

12721 GGGGCGGAGC CTATGGAAAA ACGCCAGCAA CGCGGCCTTT TTACGGTTCC TGGCCTTTTG

12781 CTGGCCTTTT GCTCACATGT AATGTGAGTT AGCTCACTCA TTAGGCACCC CAGGCTTTAC

12841 ACTTTATGCT TCCGGCTCGT ATGTTGTGTG GAATTGTGAG CGGATAACAA TTTCACACAG

12901 GAAACAGCTA TGACCATGAT TACGCCAAGC TACTCCAACG CGTTGGGAGC TCTCCCATAT

12961 GGTCGACCTG CAGGCGGCCG CGAATTCACT AGTGATTGAC CGTGACGTCA

Figure S1. Structure and sequence of CD18 gene locus exon 3 targeting vector B044.2. B044.2 consists of the pGEM-T Easy vector (Promega, Madison, WI). The CD18 gene locus encompassing insert was derived from the BAC clone RP23-95M21 (222.chori.org/bacpac). Part of intron 2 and total exon 3 were cloned into the AgeI site of pGEM-T Easy. The FRT-neomycin resistance-SV40-FRT cassette was cloned downstream of exon 3 (AgeI and HindIII/SnaBI restriction sites). Sequences comprising exons 4-6 were cloned into SnaBI site. The thymidine kinase expression cassette was cloned into the AscI site at the 3′end of exon 6. The 5′ loxP site is located upstream of exon 3 and the 3′ loxP site adjacent to the FRT-neomycin resistance-SV40-FRT cassette upstream of exon 4.

Table S1. Antibodies used for flow cytometric analysis

Antibody specificity	Clone	Fluorescence label
CD3	145-2C11, 500A2	eFl506, PE-Cy5, APC
CD4	GK1.5, RM4-5	eFl450, FITC, AF700, APC-Cy7
CD8	07.06.53	eFl450, FITC, BV510, PE-Cy5, APC-Cy7
CD11a	M17/4	PE-Cy7
CD11b	M1/70	PE, PE-Cy5, PE-Cy7
CD11c	N418	APC, PE-Cy7
CD18	M18/2	FITC, APC
CD19	1D3	PE-Cy5, SB702, APC, APC-Cy7
CD25	7D4	FITC
CD40	1C10	APC
CD45	30-F11, A20	FITC, SB702
CD62L	MEL-14	APC
CD68	FA-11	FITC, PE
CD69	H1.2F3	PE
CD80	16-10A1	PE
CD86	GL1	FITC
CD103	2,00E+007	PE
CD172a	P84	PE-Cy7
CD207	eBioL31	PE
CD317	9,27E+002	BV711
F4/80	BM8, T45-2342	e-Flour450, BB790
Foxp3	FjK-16a	FITC
GATA-3	TWAJ	PE
GR-1	RB6-8C5	APC
Ly6-C	AL-21, HK1.4	FITC, BV570, APC
Ly6-G	1A8	e-Flour450
MHCII	M5/114.15.2	eFl450, PE-Cy5
NK1.1	PK136	PE-Cy5, APC
phospho-STAT1	A15158B	PE
phospho-STAT3	13A3-1	AF488
phospho-STAT5	12-9010-42	PE
ROR-γt	AFKJS-9	APC
T-bet	eBio4B1	PerCP-Cy5.5
XCR1	ZET	BV650

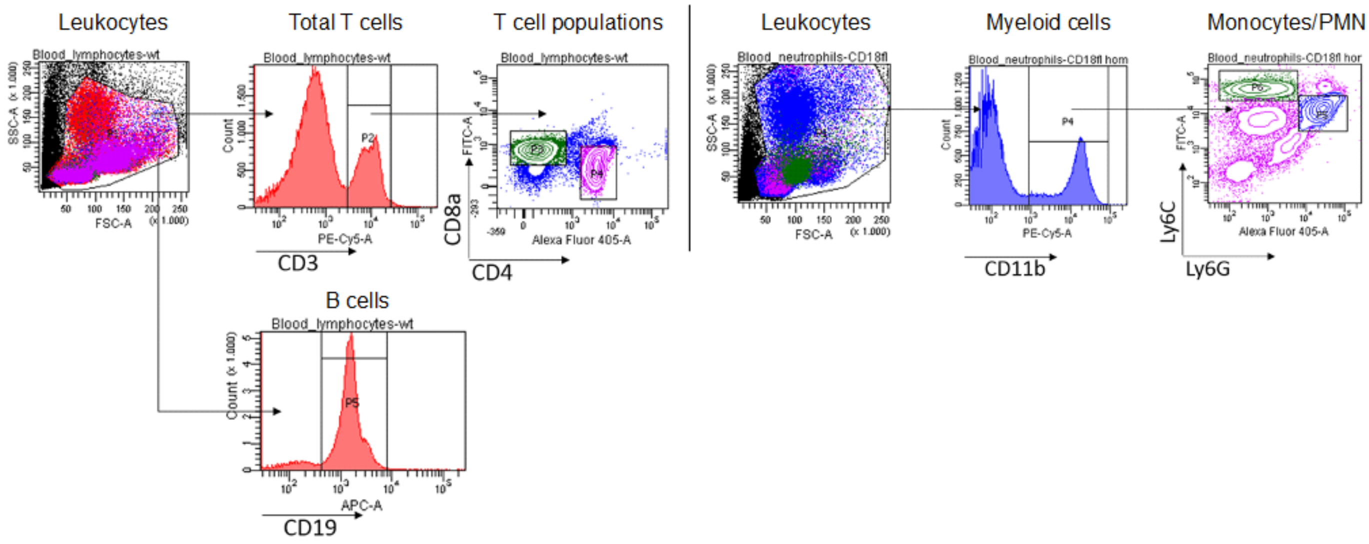

Figure S2. Gating strategy of leukocyte populations in blood (Attune NxT flow cytometer).

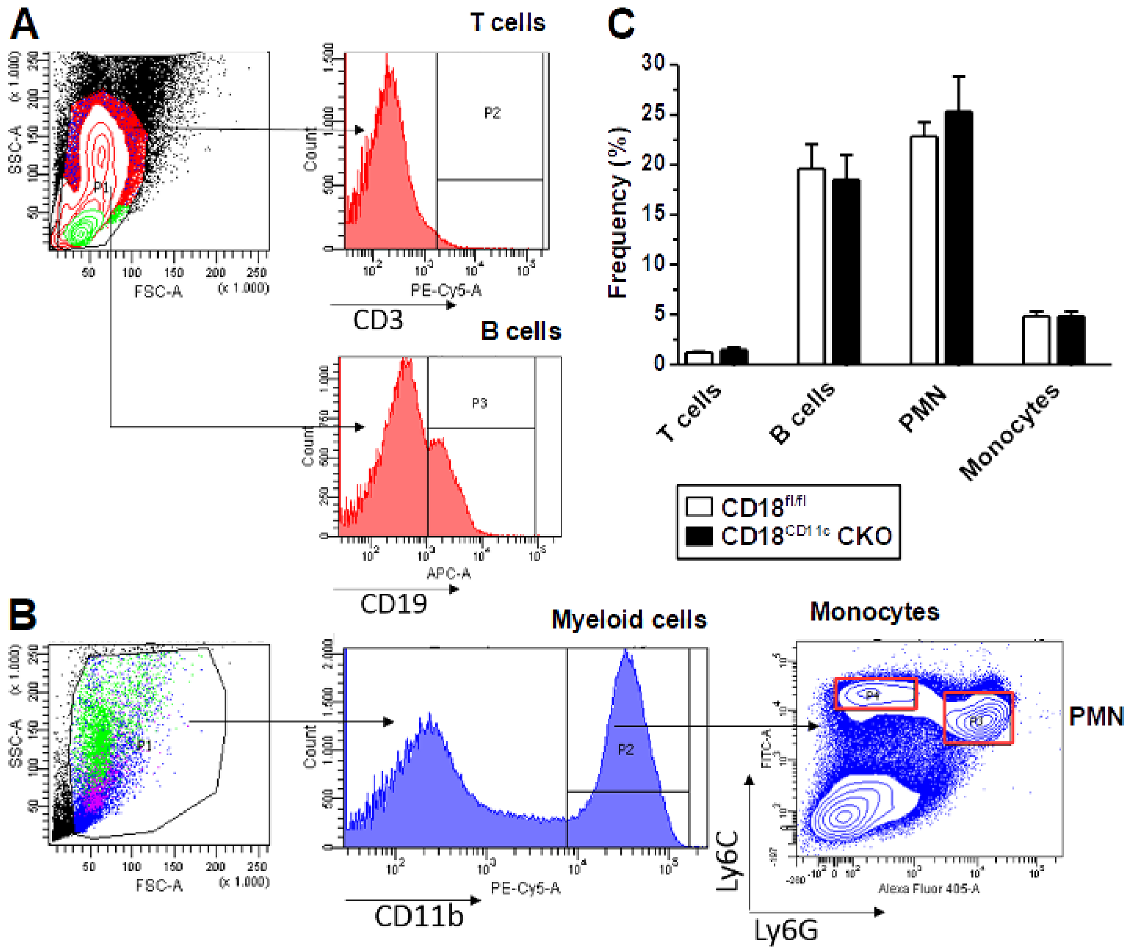

Figure S3. Frequencies of leukocyte populations in bone marrow are unaltered in CD18^{CD11c} cKO mice. (**A**, **B**) Gating strategies of (**A**) B cells and T cells as well as of (**B**) polymorphonuclear granulocytes [PMN] and monocytes as assessed by flow cytometry are shown. (**C**) Frequencies of either cell type in bone marrow are shown (mean±SEM of 4 experiments).

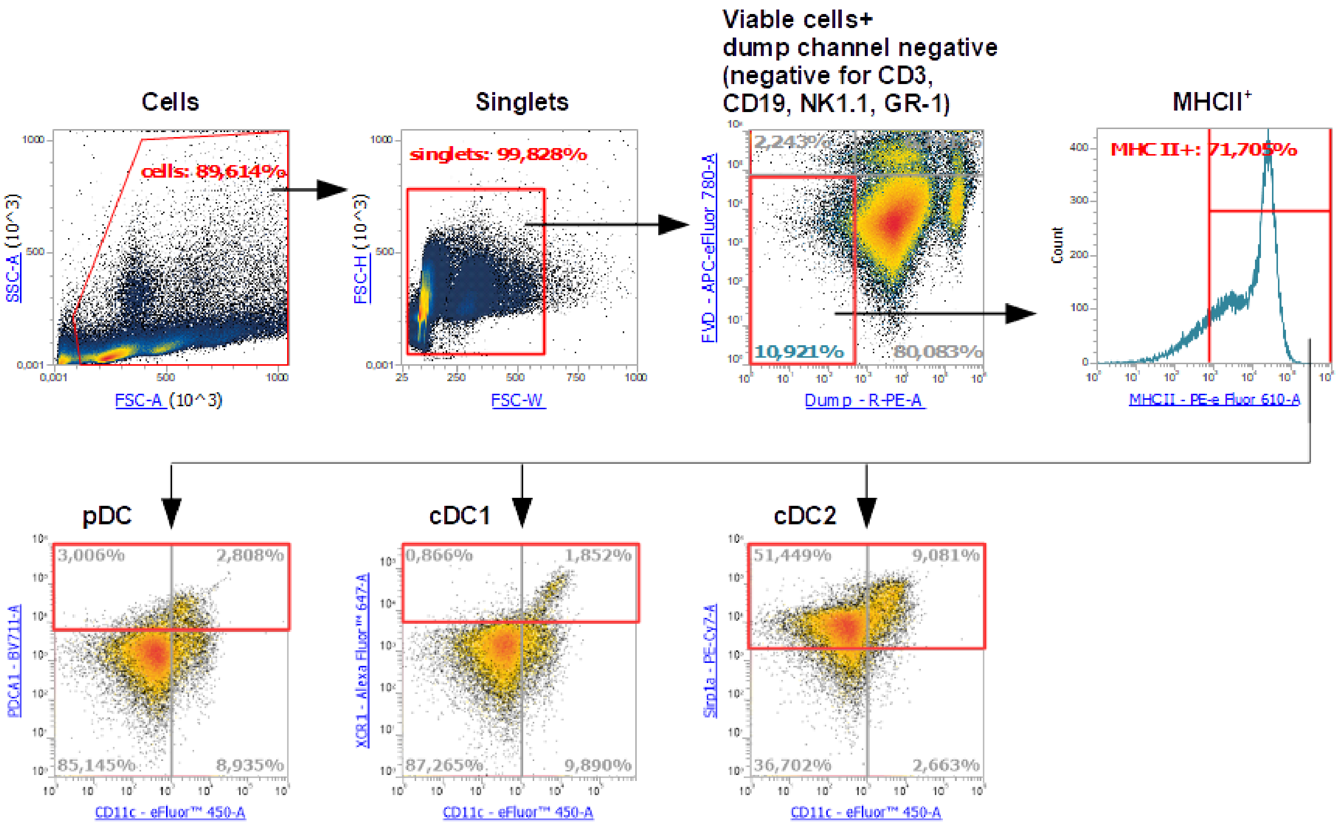

Figure S4. Gating strategy for splenic DC populations (Attune NxT flow cytometer).

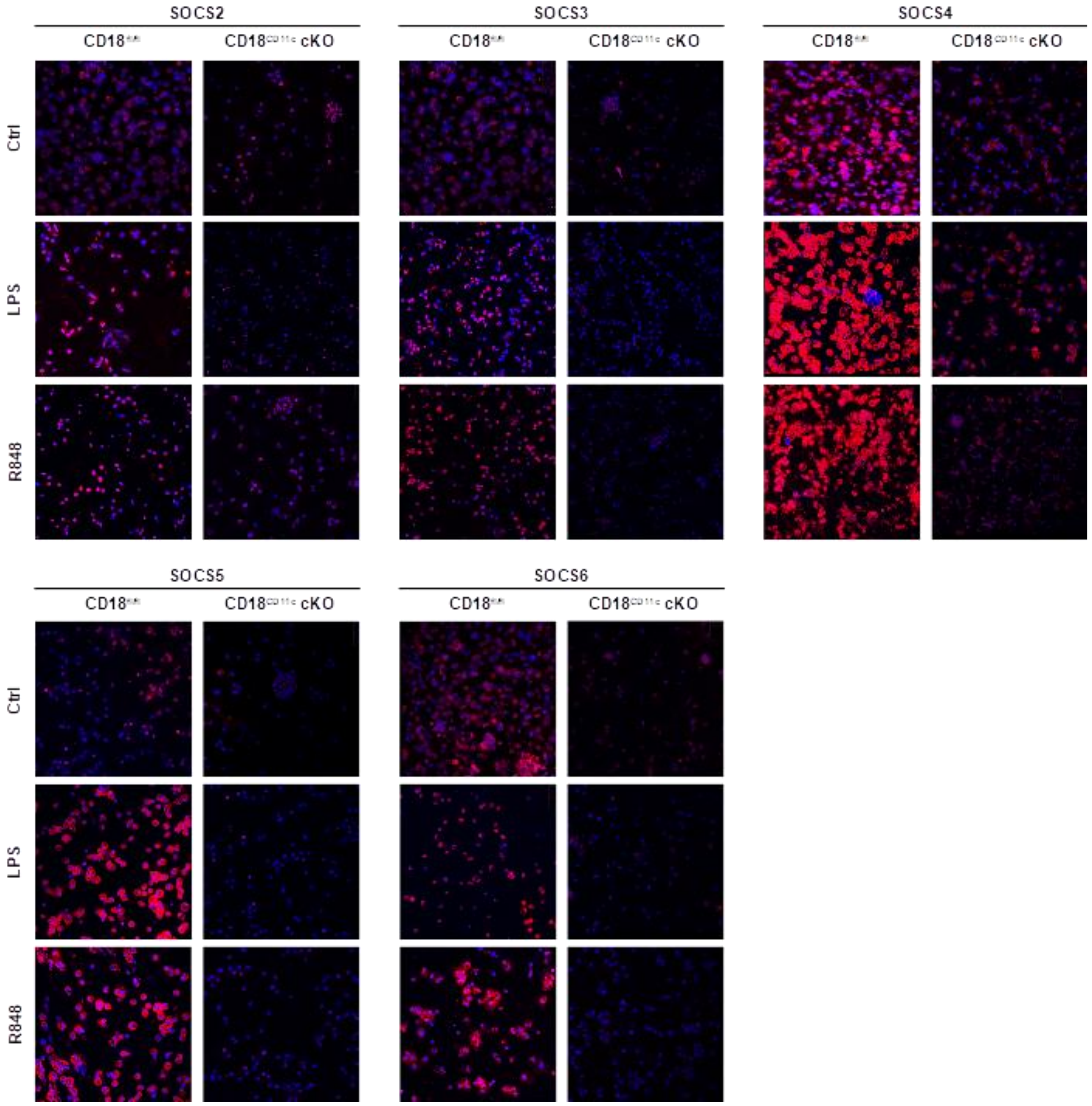

Figure S5. BMDC lacking β2 integrins display disturbed regulation of SOCS protein expression. BMDC (CD18$^{fl/fl}$, CD18^{CD11c} cKO) were stimulated overnight in parallel with LPS and R848 or were left untreated (Ctrl). On the next day, SOCS protein expression was assessed on cytospins by immunofluorescence (AF647; red) and nuclei were stained with Hoechst dye (blue) (experimental details given in 2.10.). Pictures are representative of 3-4 experiments each.

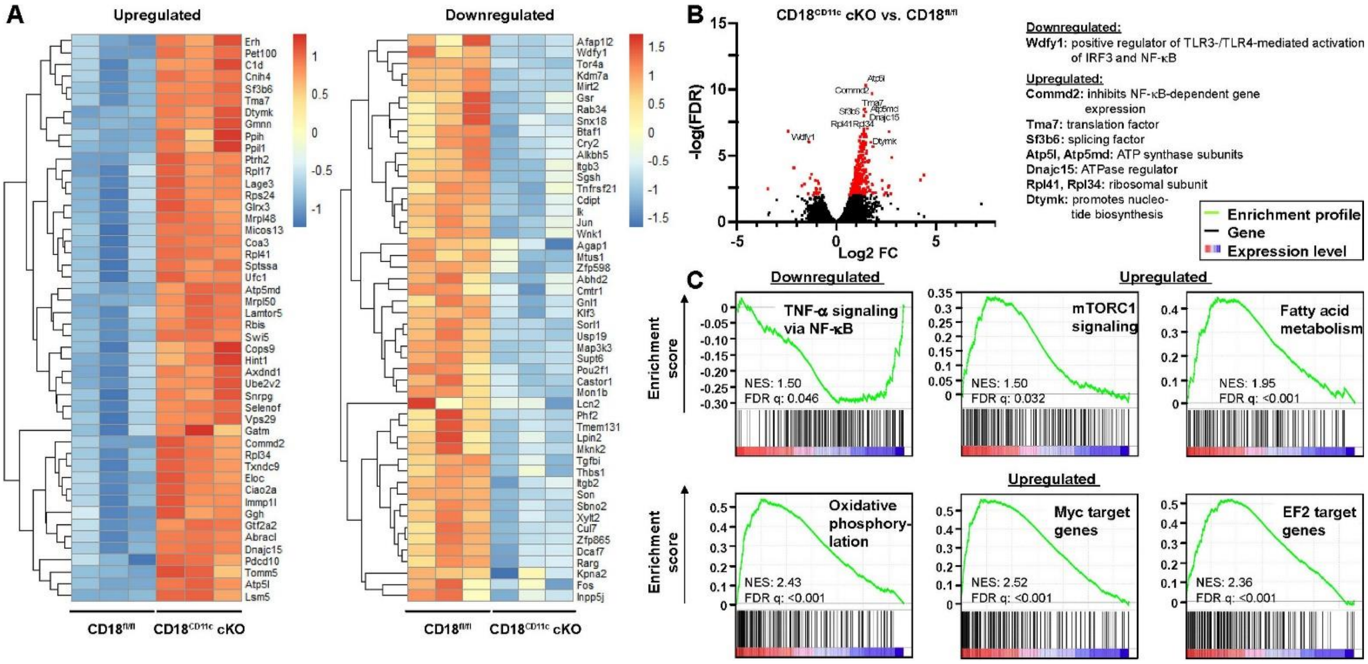

Figure S6. Downregulation of β2 integrins in BMDC results in attenuated expression of genes associated with inflammatory signaling and upregulation of genes involved in metabolic pathways, and transcription factor Myc/EF2 targets. Unstimulated BMDC ($CD18^{fl/fl}$, $CD18^{CD11c}$ cKO, each n=3) were subjected to RNA-seq analysis. (A) Heatmap representation of the Top 50 genes significantly upregulated (left panel) and downregulated (right panel) in CD18CD11c cKO versus $CD18^{fl/fl}$ BMDC clustered by hierarchical clustering. The color legend denotes the level of gene expression (low: blue, high: red) and represents z-scores. (B) Left panel: Volcano plot of all quantified mRNA species. Significantly regulated genes (t-test q-value < 0.05 and log2[fold-change] > 2) are given in red. Top 10 genes are named. Right panel: Functional role of Top 10 genes. (C) Gene set enrichment plots of significantly regulated pathways (FDR adjusted q-values <0.05). The normalized enrichment score (NES) and FDR q-values are given.

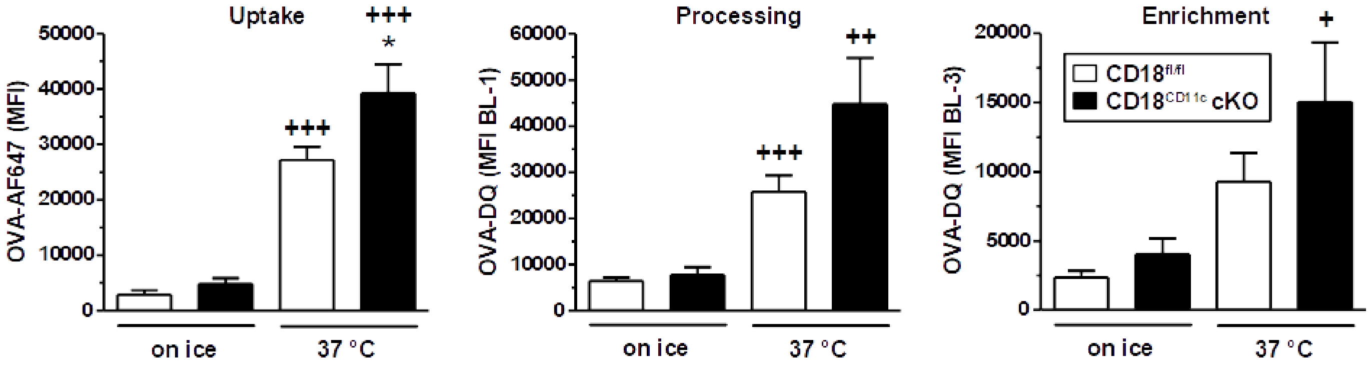

Figure S7. β2 integrin-deficient BMDC display higher antigen uptake and processing capacity. BMDC were kept on ice or at 37 °C, and OVA derivatives (OVA-AF647, OVA-DQ; each 25 µg/ml) were applied for 1h. OVA uptake (left panel), processing (middle panel) and endo-/lysosomal enrichment of processed OVA in MHCII-expressing cells were assessed by flow cytometry. Data denote the MFI (mean±SEM of 6-9 experiments). Statistical differences versus *$CD18^{fl/fl}$ and $^{+}$versus according control group (on ice) are indicated (one way ANOVA, Tukey test). $^{*,+}$p<0.05, $^{++}$p<0.01, $^{+++}$p<0.001.

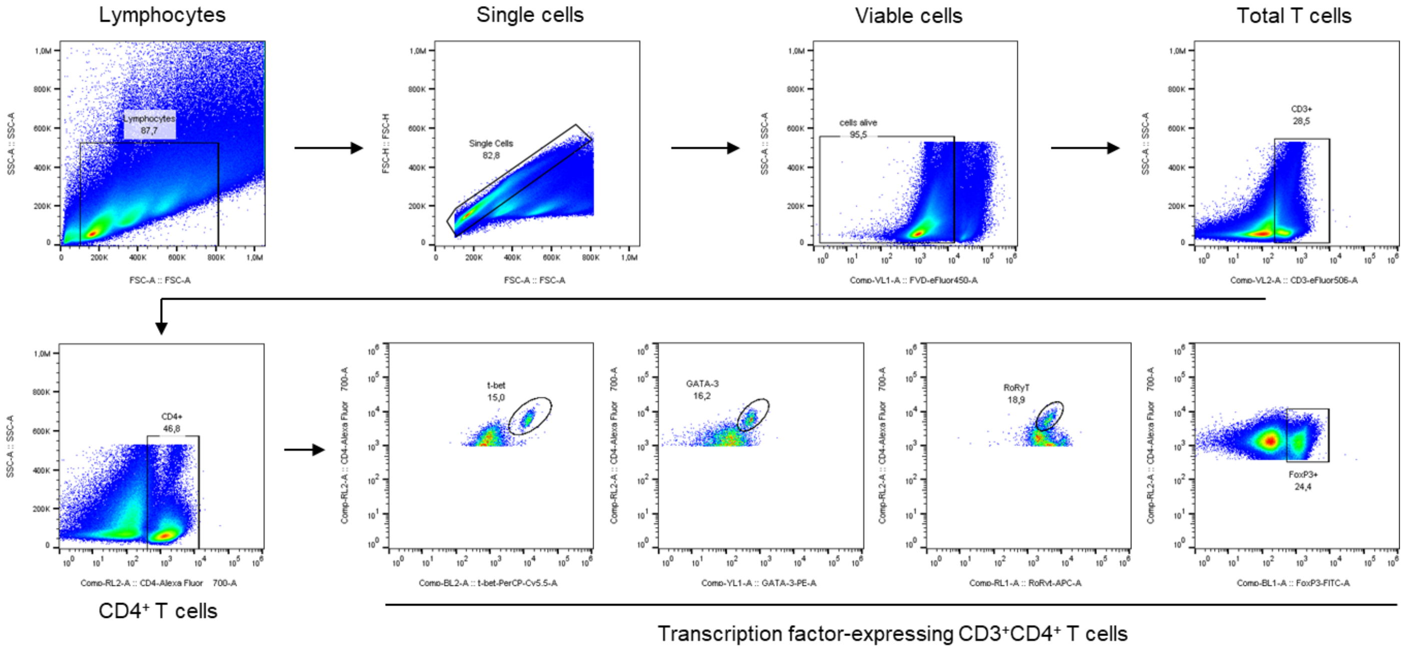

Figure S8. Gating strategy for intracellular detection of transcription factors in CD4⁺ T cells in spleen, lymph node and spinal cord (FACSymphony).

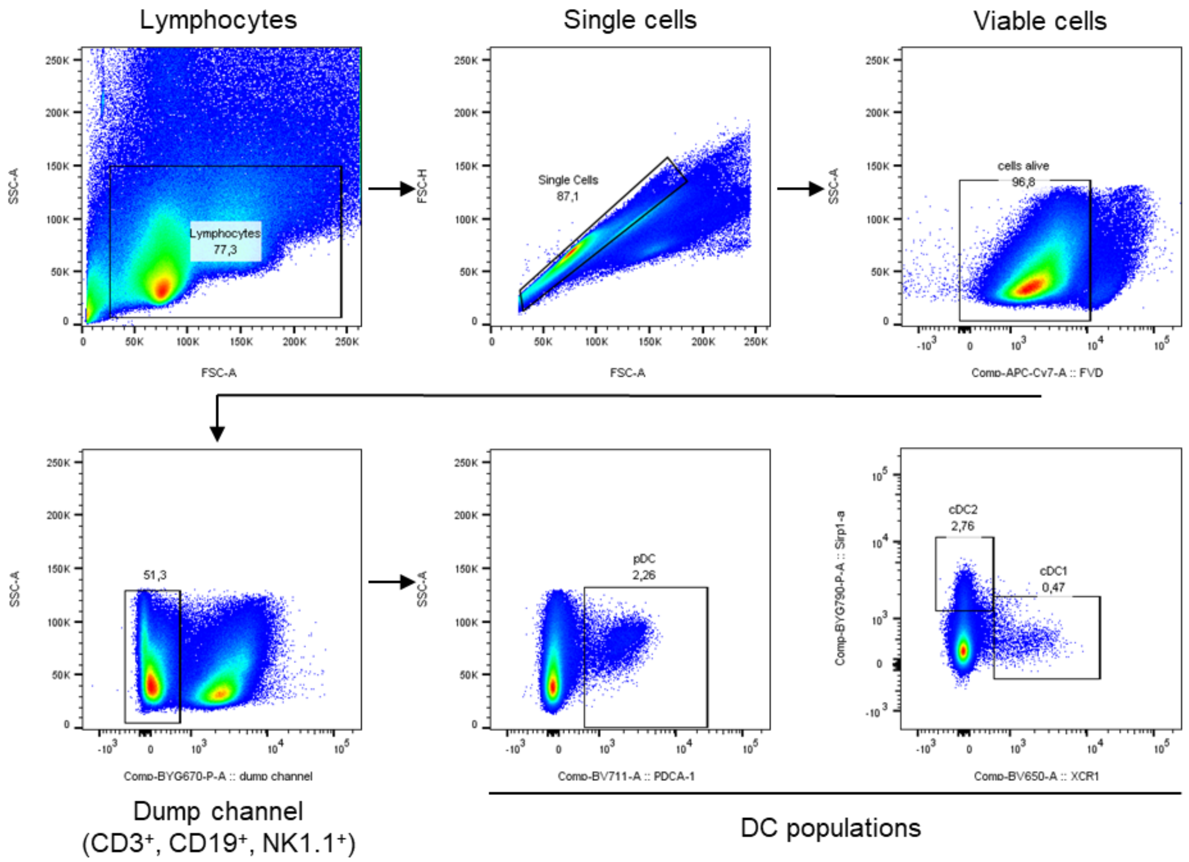

Figure S9. Gating strategy for detection of DC populations in spleen, lymph node and spinal cord (FACSymphony).

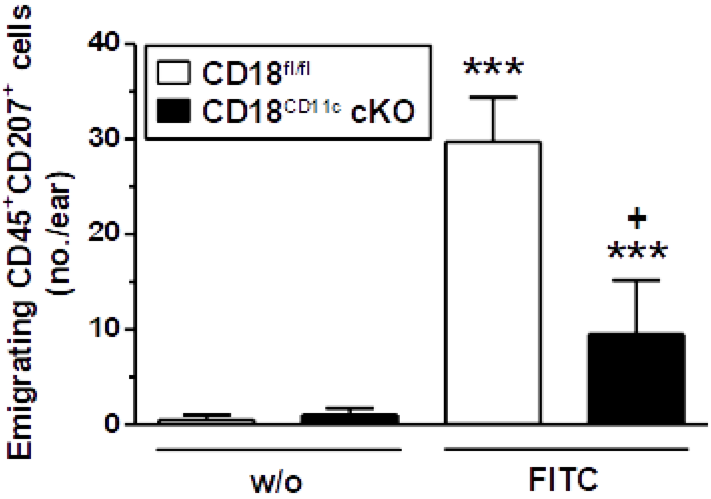

Figure S10. CD207+ DC of CD18CD11c cKO mice emigrate at lower extent from ears after FITC application. Mouse ears were split, both halves were placed into 12-well plates (1 ml of IMDM-based culture media) with the outside upwards. Then, 5 µl of FITC solution (fluorescein isothiocyanate, 5 mg/ml) dissolved in 1:1 (v/v) acetone:dibutylphalate (all from sigma-Aldrich) were applied to half of all samples. On the next day, frequencies of CD45+CD207+ DC in culture media was assessed by flow cytometry (mean ±SEM, n=4). Statistical differences versus *untreated (w/o) and +CD18fl/fl are indicated (one way ANOVA, Tukey test). +p<0.05, ***p<0.001.

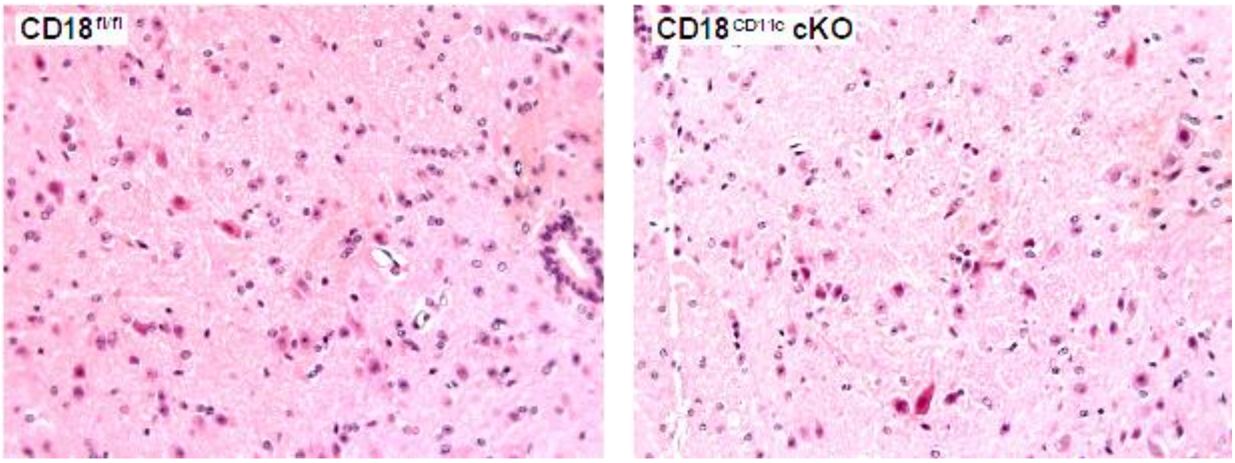

Figure S11. Spinal cord of CD18CD11c cKO mice displays somewhat lower leukocyte infiltration. Spines were retrieved on d38 after the onset of EAE and stored in 10% paraformaldehyde for histopathological analysis. Paraffin-embedded blocks were prepared, and derived sections (5 µm) were stained with hematoxylin and eosin (H&E) to assess infiltration of leukocytes. For this, H&E-stained sections were examined microscopically using a BX40 microscope equipped with a CCD camera (both from Olympus, Hamburg, Germany) for total cells per field of view (CD18fl/fl: 338; CD18CD11c cKO: 264). Pictures are representative of samples obtained from two animals each.
